# The Therapeutic Effects of the Internal Administration of Hot Water in the Treatment of Nervous Diseases

**Published:** 1884-12

**Authors:** 


### THE THERAPEUTICAL EFFECTS OF THE INTERNAL ADMINISTRATION OF HOT WATER IN THE TREATMENT OF NERVOUS DISEASES.

Dr. A. L. Ranney read a paper with the above title, in which he maintained that the benefits following the internal use of hot water were due almost entirely to heat, and gave the following rules for its administration:

1. An ordinary goblet contains about ten ounces, and the quantity may be from one to one and a half goblet.
2. The water may be flavored with lemon, etc.; fifteen minutes may be consumed in sipping a gobletful; wooden cups prevent it from cooling quickly; and it must be taken *hot and not warm*—from 110° to 150° F.

3. It must be taken one hour and a half before each meal, with absolute punctuality, and at bed-time.

4. Increase the temperature of the water as fast as the patients can bear it.

5. The administration of hot water must be continued for at least six months to get its full effects.

6. The dose should be determined largely by the specific gravity and general character of the urine. The object is to bring the specific gravity of the urine to the standard of health.

7. The use of cold fluids in the form of beverages must be absolutely prohibited.

8 Constricted diet is often necessary to the full effects of the treatment in some form of nervous derangements.

The above extract from recently published proceedings of the New York Academy of Medicine* may be regarded as a quasi professional indorsement of one of the popular fallacies of the day. Almost every year revives some old, or brings to public notice some new, specific or method of treating the class of diseases above mentioned. Each in its turn is extravagantly praised, and long lists of cases relieved or cured by it thrust upon public attention, each to be succeeded by some further or more startling expedient. The whey cure, the grape cure, the meat cure and the mud cure, and thousands more, have had their brief period of reputation. Dry sand taken in spoonful doses has had its advocates. Kneading, friction, inunction, starvation and repletion have found advocates. Brown bread, oatmeal, mush, hot milk, especially ass's milk, haye been often tried, the last perhaps with good reason, if the maxim "*similia similibus curantur*" be of universal application.

It is undeniable that after the use of any of those and many others of like character and temporary repute amendments or cures have been noted. Judged by the number of certificates or the character of the certifiers, no one of them can claim superiority over the others. Ignoring the fact that spontaneous cures in this class of dis-

*New York Medical Record, Oct. 25th.

eases are very common, and the potent agencies of change of habits and regimen which usually accompany their employment, their advocates assume that the improvement or recovery is the direct effect of the popular nostrum last used.

The absurdity of this assumption is apparent. The attempts made to explain the assumed good results are among the most amusing of the efforts of so-called medical literature. Those by which Dr. Ranney accompanied the paper from which the above paragraphs are taken are quite as noteworthy as their kindred in former times; in fact they invite criticism did time and opportunity permit; but our purpose is only to spread before our patrons the exact "rules" for the administration of this remedy.

It is true that the remedy is not exactly new; we certainly have heard of it before. If we are not mistaken a renowned practitioner in some foreign country (Sangrado was the name of him), gave it extensive trial with more or less of success. He is supposed to have practiced blood-letting conjointly with hot water, suggesting a doubt whether the remarkable statistics of his practice were due to one or the other. Hereafter this doubt will be removed, and the practice of medicine simplified, its whole armamentaria will consist of a tea kettle and a graduate measure.

